# Complete Genome Sequence of *Elizabethkingia miricola* Strain EM798-26 Isolated from the Blood of a Cancer Patient

**DOI:** 10.1128/genomeA.01408-17

**Published:** 2018-01-04

**Authors:** Jiun-Nong Lin, Chung-Hsu Lai, Chih-Hui Yang, Yi-Han Huang, Hsi-Hsun Lin

**Affiliations:** a Division of Infectious Diseases, Department of Internal Medicine, E-Da Hospital, I-Shou University, Kaohsiung, Taiwan; b Department of Critical Care Medicine, E-Da Hospital, I-Shou University, Kaohsiung, Taiwan; c School of Medicine, College of Medicine, I-Shou University, Kaohsiung, Taiwan; d Department of Biological Science and Technology, Meiho University, Pingtung, Taiwan

## Abstract

*Elizabethkingia miricola* EM798-26 was isolated from the blood of a patient with diffuse large B-cell lymphoma in Taiwan. We report here the complete genome sequence of EM798-26, which contains a G+C content of 35.7% and 3,877 candidate protein-coding genes.

## GENOME ANNOUNCEMENT

*Elizabethkingia* is a genus of aerobic, Gram-negative, nonmotile, non-spore-forming, nonfermenting bacilli that belongs to the family *Flavobacteriaceae* (1). The genus *Elizabethkingia* previously included three species: *E. meningoseptica*, *E. miricola*, and *E. anophelis* (1). Recently, three novel species, namely, *E. bruuniana* sp. nov., *E. ursingii* sp. nov., and *E. occulta* sp. nov., were proposed (2). These microorganisms have rarely been reported to cause diseases in humans. However, the case fatality rate of *Elizabethkingia* infections ranges from 24% to 60%, particularly in immunocompromised patients (3, 4).

*E. miricola* strain EM798-26 was isolated from the blood of a patient with diffuse large B-cell lymphoma in Taiwan. This patient suffered from neutropenic fever after chemotherapy for the lymphoma. This isolate was identified as *E. miricola* according to the results of 16S rRNA gene sequencing (5). The genome of *E. miricola* EM798-26 was sequenced using an Illumina HiSeq 2000 sequencing platform (Illumina, San Diego, CA, USA), and the short reads were assembled using SOAPdenovo version 2.04 (6). The reads produced by PacBio’s long-read sequencing technology (Pacific Biosciences, Menlo Park, CA, USA) were assembled using the RS_HGAP_Assembly3 protocol in SMRT Analysis version 2.2.0 (7). The hybrid genome sequenced by the HiSeq and PacBio platforms was then assembled using Rabbit software (8). The structural errors were corrected by optical mapping (Bionano Genomics, San Diego, CA, USA). The assembled genome was submitted to NCBI Prokaryotic Genome Annotation Pipeline for gene annotation (9).

The total length of the assembled genome was 4,393,011 bp, with a mean G+C content of 35.7%. The genome coverage was 220.0×. The genome contained 4,029 genes and 3,957 coding sequences. There were 3,877 protein-coding genes and 80 pseudogenes. The number of RNA genes was 72, including 15 rRNAs (5S, 5; 16S, 5; 23S, 5), 54 tRNAs, and 3 noncoding RNAs.

Since its first identification in 2003 from condensation water on the space station Mir collected in 1997, *E. miricola* has been sporadically reported to cause human infections, including bacteremia, sepsis, pneumonia, pulmonary abscess, arthritis, and neutropenic fever (10–13). However, studies on *E. miricola* are still limited. Knowledge of the genome sequence of *E. miricola* will provide researchers with valuable information to understand this lethal microorganism.

### Accession number(s).

This complete genome sequence has been deposited at GenBank under the accession number CP023746.
